# Cannabinoid Receptors Modulate Neuronal Morphology and AnkyrinG Density at the Axon Initial Segment

**DOI:** 10.3389/fncel.2017.00005

**Published:** 2017-01-25

**Authors:** Mónica Tapia, Ana Dominguez, Wei Zhang, Ana del Puerto, María Ciorraga, María José Benitez, Carmen Guaza, Juan José Garrido

**Affiliations:** ^1^Instituto Cajal, Consejo Superior de Investigaciones Científicas (CSIC)Madrid, Spain; ^2^Department of Quimica Fisica Aplicada, Universidad Autónoma de MadridMadrid, Spain

**Keywords:** axon initial segment, cannabinoids, CB1R, 2-AG, ankyrinG, dendrites, axon, hippocampal neurons

## Abstract

Neuronal polarization underlies the ability of neurons to integrate and transmit information. This process begins early in development with axon outgrowth, followed by dendritic growth and subsequent maturation. In between these two steps, the axon initial segment (AIS), a subcellular domain crucial for generating action potentials (APs) and maintaining the morphological and functional polarization, starts to develop. However, the cellular/molecular mechanisms and receptors involved in AIS initial development and maturation are mostly unknown. In this study, we have focused on the role of the type-1 cannabinoid receptor (CB1R), a highly abundant G-protein coupled receptor (GPCR) in the nervous system largely involved in different phases of neuronal development and differentiation. Although CB1R activity modulation has been related to changes in axons or dendrites, its possible role as a modulator of AIS development has not been yet explored. Here we analyzed the potential role of CB1R on neuronal morphology and AIS development using pharmacological and RNA interference approaches in cultured hippocampal neurons. CB1R inhibition, at a very early developmental stage, has no effect on axonal growth, yet CB1R activation can promote it. By contrast, subsequent dendritic growth is impaired by CB1R inhibition, which also reduces ankyrinG density at the AIS. Moreover, our data show a significant correlation between early dendritic growth and ankyrinG density. However, CB1R inhibition in later developmental stages after dendrites are formed only reduces ankyrinG accumulation at the AIS. In conclusion, our data suggest that neuronal CB1R basal activity plays a role in initial development of dendrites and indirectly in AIS proteins accumulation. Based on the lack of CB1R expression at the AIS, we hypothesize that CB1R mediated modulation of dendritic arbor size during early development indirectly determines the accumulation of ankyrinG and AIS development. Further studies will be necessary to determine which CB1R-dependent mechanisms can coordinate these two domains, and what may be the impact of these early developmental changes once neurons mature and are embedded in a functional brain network.

## Introduction

The establishment of a functional brain network depends on the ability of neurons to reach and activate their specific targets via the growth and elongation of their axons. Axonal outgrowth is the first morphological event of neuronal polarization, followed by the development of dendrites and axon initial segment (AIS) formation (Ramón Y Cajal, 1897; Kaech and Banker, 2006). The AIS plays an essential role in maintaining neuronal polarity and axon integrity and identity (Hedstrom et al., 2008). Functionally, the AIS is a unique neuronal domain comprising high densities of Na^+^, K^+^ and Ca^2+^ voltage-gated ion channels (for a review see Bender and Trussell, 2012). These voltage-gated ion channels are anchored in the AIS by interactions with scaffold cytoskeletal proteins, such as ankyrinG or PSD-93 (Garrido et al., 2003; Pan et al., 2006; Ogawa et al., 2008). Among other factors, the high density of ion channels confers on the AIS the property of being the site of action potential (AP) initiation (Stuart et al., 1997; Kole et al., 2008). In fact, the AIS acts as an electrical gatekeeper, becoming the decision point for initiation of APs. The AIS performs this function by summing all excitatory and inhibitory inputs from thousands of synapses until a critical firing threshold is reached and the AP is irreversibly triggered. The scaffold protein ankyrinG is the AIS master regulator and is necessary for its assembly and maintenance, such that ankyrinG suppression leads to a loss of axonal identity characterized by the appearance of dendritic spines in former axonal structures (Hedstrom et al., 2008; Sobotzik et al., 2009). Over the past 20 years, many studies have contributed to describe AIS molecular structure, function and some regulatory mechanisms (some examples and reviews are: Kobayashi et al., 1992; Zhou et al., 1998; Garrido et al., 2003; Rasband, 2010; Grubb et al., 2011; Sanchez-Ponce et al., 2011; Bender and Trussell, 2012; Zollinger et al., 2015; Huang and Rasband, 2016). Furthermore, the discovery of AIS structural plasticity (changes in position or length) and its capacity to adapt in response to different physiological and pathological conditions in order to maintain neuronal survival and excitability shed light on the dynamical complexity of the AIS (Schafer et al., 2009; Grubb and Burrone, 2010; Kuba et al., 2010). Besides AIS structural plasticity, AIS proteins density also can change in response to physiological or pathological stimuli (Kuba et al., 2015; del Puerto et al., 2015).

However, it is now important to understand which neuronal or glial mechanisms, and which membrane receptors, contribute to different types of AIS modulation. Moreover, the mechanisms involved in the targeting and modulation of AIS proteins during the very early stages of neuronal development remain elusive. Recent studies have shown that the purinergic system (del Puerto et al., 2015), serotonin receptors (Ko et al., 2016) or GABAergic innervation (Muir and Kittler, 2014) influence and modulate AIS proteins and neuronal excitability. However, the role of another important neuromodulatory system, the cannabinoid system, has not been analyzed in the context of AIS development and maturation.

The endocannabinoid system (ECS) comprises endocannabinoids (eCB) such as 2-arachidonoyl glycerol (2-AG) and n-arachidonoyl ethanolamine (AEA or anandamide), the enzymes responsible for their synthesis/degradation and the cannabinoid receptors. Among cannabinoid receptors, type-1 cannabinoid receptor (CB1R) is abundantly expressed in the central nervous system, particularly in the cortex, basal ganglia, hippocampus and cerebellum (Mackie, 2005), and is considered one of the most abundant G protein-coupled receptors (GPCRs) expressed in the brain (Matsuda et al., 1990; Kano et al., 2009). CB1R coupling to Gi/o proteins leads to multiple downstream events depending on the cell type: adenylyl cyclase inhibition, voltage-gated calcium channel inhibition, activation of inwardly rectifying potassium channels and activation of mitogen-activated protein kinases (Howlett, 2005). Despite the lack of highly specific pharmacology for the study of cannabinoid receptors, several studies have suggested that CB1Rs are mostly present in axon terminals, and also in astrocytes (Navarrete and Araque, 2010), and control a wide spectrum of physiological and pathological conditions (Katona and Freund, 2012; Mechoulam and Parker, 2013). The type-2 cannabinoid receptors (CB2Rs) are considered the predominant cannabinoid receptor in the immune system and their neuronal expression has only been described under certain pathological conditions (Viscomi et al., 2009). Besides their well-known functions as neuromodulators, controlling both neuronal excitability and synaptic plasticity at different scales (Chevaleyre et al., 2006; Kano et al., 2009; Marinelli et al., 2009; Katona and Freund, 2012), eCB also seem to be involved in brain development and axonal pathfinding (Mulder et al., 2008; Keimpema et al., 2011). Indeed, CB1Rs are highly expressed in the developing brain (Vitalis et al., 2008), and axon development and pathfinding are disrupted during pre- and post natal brain development in CB1R knock out mice (Mulder et al., 2008; Wu et al., 2010). CB1Rs are also involved in the regulation of adult neurogenesis, such that their loss inhibits neuronal progenitor cell proliferation *in vivo* and *in vitro*. Moreover CB1R expression in neural progenitors increases along neuronal differentiation, allowing eCBs to control neuronal specification and morphogenesis (reviewed in Galve-Roperh et al., 2013).

Thus, a tempting idea is that cannabinoid receptors play an important role during the early stages of neuronal domain development and maturation. In this article, we will focus on new data concerning the role of the cannabinoid system in the modulation of AIS and its relation with axonal and dendritic development. We demonstrate a differential neuronal CB1R modulation of axonal and dendritic development, as well as a role of CB1R on the modulation of ankyrinG density at the AIS at different early developmental stages. Moreover, our data suggest that glial CB2Rs also participate in ankyrinG density maintenance.

## Materials and Methods

### Reagents and Plasmids

2-AG, AM-251, AM630 and SR141716A were obtained from Tocris. UCM-03025 was a gift from María Luz Lopez Rodriguez laboratory (Complutense University, Madrid; Hernández-Torres et al., 2014). CB1R interference RNA and scrambled RNA plasmids (Origene) were a kind gift of Dr. Ismael Galve-Ropert (Universidad Complutense, Madrid) and were previously validated (Diaz-Alonso et al., 2012).

### Animals

Animals were housed in a room at controlled temperature and relative humidity with alternating 12 h light and dark cycles and free access to food and water “*ad libitum*”. Animal care protocols used in our laboratory are in conformity with the appropriate national legislation (53/2013, BOE no. 1337) and guidelines of the Council of the European Communities (2010/63/UE). All protocols were previously approved by the CSIC bioethics committee.

### Neuronal Culture

Mouse hippocampal neurons were prepared as previously described (del Puerto et al., 2012, 2015). Neurons were obtained from E17 mouse hippocampi, which were incubated in a 0.25% trypsin solution in Ca^2+^/Mg^2+^ free Hank’s buffered salt solution (HBSS) and dissociated using fire-polished Pasteur pipettes. The cells were plated on polylysine-coated coverslips (1 mg/mL) at a density of 5000 cells/cm^2^ for 2 h in plating medium (minimum essential medium [MEM], 10% horse serum, 0.6% glucose, Glutamax-I and antibiotics). Then coverslips were inverted and transferred to culture dishes containing astrocytes. Astrocyte medium was replaced by neuronal culture medium 24 h before neuronal culture (Neurobasal medium, B27 supplement, Glutamax-I). To avoid contact between neurons and astrocytes, paraffin beads were placed on coverslips before neuronal plating. 5 μM 1-β-D-arabinofuranosylcytosine (AraC) was added after 2 days in culture to avoid glial proliferation. Pharmacological treatments were applied as described in the “Results” Section. In the case of pharmacological treatments in the absence of glial cell layer, coverslips were transferred to plates containing glial cell-conditioned medium. Primary hippocampal neurons were nucleofected using the Amaxa nucleofector kit for primary mammalian neural cells (Amaxa Bioscience) according to the manufacturer’s instructions. Nucleofection was performed using 3 μg of total DNA and 3 × 10^6^ cells for each nucleofection. Neurons were plated at a density of 10,000 cells/cm^2^ as described above. Nucleofection efficiency was ~15% of neurons, based on the number of GFP-positive neurons. For Western-blot experiments, neurons were plated at a density of 50,000 cells/cm^2^ and processed as previously described (Tapia et al., 2013).

### Immunofluorescence

Neurons were fixed in 4% PFA, then coverslips were treated for 10 min with 50 mM NH4Cl and incubated in blocking buffer (0.22% gelatin, 0.1% Triton X-100 in PBS) for 30 min, before incubation with primary antibodies for 1 h at room temperature in blocking buffer. For CB1R staining, neurons were incubated for 30 min at 37°C with anti-CB1R antibody, then rinsed three times and fixed to continue the procedure with other antibody staining. The primary antibodies used were: chicken anti-MAP2 (1:10,000, Abcam), mouse anti-ankyrinG (1:100) from NeuroMab, anti-Tau-1 from Millipore (1:1000) and rabbit anti-CB1R (1:50) from Cayman (Cat. 101500). The secondary antibodies used were a donkey anti-mouse, anti-rabbit or anti-chicken Alexa-Fluor 488, 594, or 647 (1:1000). Phalloidin Alexa-Fluor 594 was used at a concentration of 1:100. Nuclei were stained using 4′,6-diamidino-2-phenylindole, and coverslips were mounted in Fluoromount G. Images were acquired on a vertical Axioskop-2 plus microscope (Zeiss) or a Leica SP5 confocal microscope under the same conditions to compare intensities. Figures were prepared for presentation using the Adobe CS4 software.

### Dendrites, Axon and AIS Measurements

Quantification of fluorescence intensity at the AIS was performed in neurons from at least three independent experiments. Measurements of ankyrinG fluorescence intensity were performed on confocal images. Using ImageJ software we drew a line starting at the limit of neuronal soma identified by MAP2 staining, and extended it along the ankyrinG staining or the GFP signal of the axon. Data from every 0.16 μm along the first 40 μm were obtained and smoothed using the Sigmaplot software to obtain average ankyrinG fluorescence intensity every 1 μm. Data were normalized in each neuron considering the value of maximum mean fluorescence in control neurons to be 100%. Total fluorescence intensity for each neuron was obtained by adding ankyrinG fluorescence values from 0 μm to 40 μm. AIS start, end and maximum fluorescence intensity were determined following the criteria described in Grubb and Burrone (2010). Taking 100% fluorescence as the maximum fluorescence intensity point, start and end points were defined as the points were fluorescence intensity is lower than 33%.

Dendrite and axon lengths were obtained based on MAP2 or Tau-1 staining using NeuronJ software to measure the length of dendritic arbor in each neuron or the axonal length including ramifications. In order to study correlations between dendrites and AIS fluorescence, both data were obtained from the same neurons.

### Statistical Analysis

All statistical analyses were carried out in Sigmaplot v12.5 (Systat Software Inc., San Jose, CA, USA) and Prism 6 (GraphPad Software, Inc., La Jolla, CA, USA). Data for each independent sample were obtained from at least three independent experiments. Data from each experiment were collected from at least 30 cells (between 30 and 50 cells) in each experimental condition. Statistical analysis was performed by *t*-test for two group comparisons and one-way ANOVA for multiple group comparisons. When data were non-normally distributed, non-parametric tests were used: Mann-Whitney Rank test for two independent samples and Kruskal-Wallis for analysis of multiple groups. In the analysis of multiple comparison, a *post hoc* analysis was performed using Bonferroni’s (in the case of ANOVA) or Dunn’s test (in the case of Kruskal-Wallis). All *p*-values were adjusted to account for multiple comparison. Cell-to-cell analysis of dendrite length and ankyrinG fluorescence was performed using Prism 6 and Sigmaplot v12.5 with a Pearson product moment test. Differences were considered significant when *p* < 0.05.

## Results

### CB1Rs Modulate Axonal and Dendritic Development

**Figure 1:**
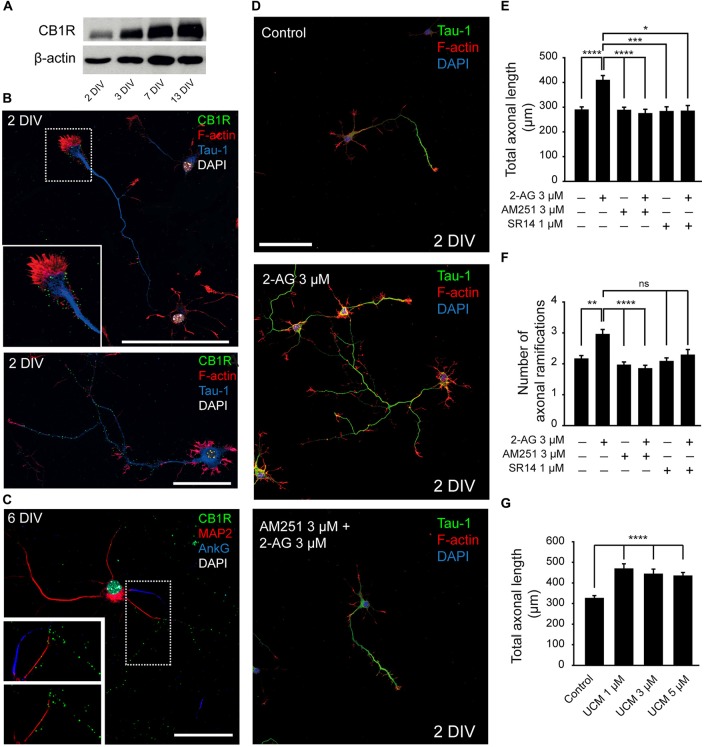
**Modulation of axonal growth by the type-1 cannabinoid receptor (CB1R). (A)** Western-blot showing CB1 receptor total expression levels on 2, 3, 7 and 13-days *in vitro* (DIV) hippocampal neurons. **(B)** CB1 receptor (green) expression in 2-DIV hippocampal neurons. Growth cones are stained with phalloidin-Alexa 594 (red), axons with Tau-1 antibody (blue) and nuclei with DAPI (white). The inset shows a magnification of the growth cone region. Note that CB1 receptors show a punctate staining and are mainly concentrated in the axonal growth cone and along the axonal shaft (image below). **(C)** CB1 receptor (green) expression in 6-DIV hippocampal neurons. CB1 receptors are expressed along the axon and are not detected by immunofluorescence in the dendrites (red) or the axon initial segment (AIS; blue). Magnification of the image is shown below with or without AIS marker staining (AnkyrinG, blue). **(D)** 2-DIV hippocampal neurons treated with 2-Arachidonylglycerol (2-AG) or 2-AG in combination with AM251 from plating until 2 days. Neurons were stained using a Tau-1 antibody and Phalloidin for better recognition of neuronal and axonal morphology. Scale bar = 100 μm. **(E–G)** Bar plots representing the mean ± SEM of axonal length or total number of axonal ramifications of 2-DIV hippocampal neurons treated with CB1R antagonists, and/or agonist **(E,F)** or treated with monoacylglycerol lipase (MAGL) inhibitor UCM03025 **(G)**. Data were acquired from three independent experiments (30 neurons/experimental condition in each experiment). Kruskal-Wallis, Dunn’s multiple comparisons test. Adjusted *p* values: *p < 0.05, **p < 0.01, ***p < 0.001, ****p < 0.0001; ns, non-significant.

In order to understand how cannabinoids may modulate neuronal development, we studied their role during axonal growth and dendrite development. First, we analyzed the expression of CB1R in cultured hippocampal neurons using a polyclonal antibody directed against the N-terminus of the receptor (Howlett et al., 1998). CB1R was detected by Western-blot after 2 days *in vitro* (2 DIV) and its expression progressively and markedly increased until 13 DIV (Figure 1A). Next, we analyzed its membrane subcellular localization by immunocytochemistry at different developmental stages. Consistent with previous reports (Irving et al., 2000), the analysis of receptor immunoreactivity revealed a punctate pattern along the axon and at the growth cone in 2-DIV neurons (Figure 1B). While the same pattern of staining was conserved in older neurons (6 DIV), no CB1R expression was detected in dendrites or in the AIS (Figure 1C). Based on this specific pattern of subcellular localization of CB1R, we sought to determine whether CB1R could be involved in axonal elongation. Hippocampal neurons were cultured since plating until 2 DIV in the presence of 2-AG (3 μM) alone or in combination with the CB1R antagonists AM251 (3 μM) or SR141716A (1 μM; Figure 1D). Axon length was increased by 40% in neurons treated with only 2-AG (408.03 ± 19.92 μm compared to 288.12 ± 12.97 μm in non-treated control neurons, Kruskal-Wallis, Dunn’s multiple comparison test, *p* < 0.0001, Figure 1E). However, treatment of neurons with two different CB1 antagonists had no effect on axonal growth (286.74 ± 13.07 μm for AM251 and 282.03 ± 19.52 μm for SR141716A vs. 288.12 ± 12.97 μm in control neurons, Kruskal-Wallis, Dunn’s multiple comparisons test, Figure 1E). In order to demonstrate that axonal elongation following 2-AG application was specifically due to CB1R activation, neurons were pre-incubated for 1 h with the CB1R antagonists AM251 or SR141716A before 2-AG application (Figure 1E). The addition of CB1R antagonists prevented the 2-AG-mediated increase in axonal elongation (273.21 ± 18.1 μm for 2-AG + AM251 and 283.28 ± 23.1 μm for 2-AG + SR141716A vs. 408.03 ± 19.92 μm in 2-AG treated neurons, *p* < 0.0001 and *p* < 0.05, and 288.12 ± 12.97 μm in control neurons, Kruskal-Wallis, Dunn’s multiple comparisons test, Figure 1E), confirming that CB1R activation promotes axonal elongation. In addition, 2-AG treatment also increased axonal ramification and, as observed for axonal elongation, this increase was prevented when neurons were pre-incubated for 1 h with the CB1R antagonists (Figure 1F). Moreover, we tested the effect of increasing endogenous levels of 2-AG by using UCM-03025, a monoacylglycerol lipase (MAGL) inhibitor that prevents 2-AG degradation (Hernández-Torres et al., 2014). Similar to what occurred when neurons were treated with 2-AG, MAGL inhibition also promoted axonal elongation (467.54 ± 25.35 μm for UCM 1 μM vs. 324.15 ± 14.42 μm in control neurons, Kruskal-Wallis, Dunn’s multiple comparisons test, *p* < 0.0001, Figure 1G).

**Figure 2:**
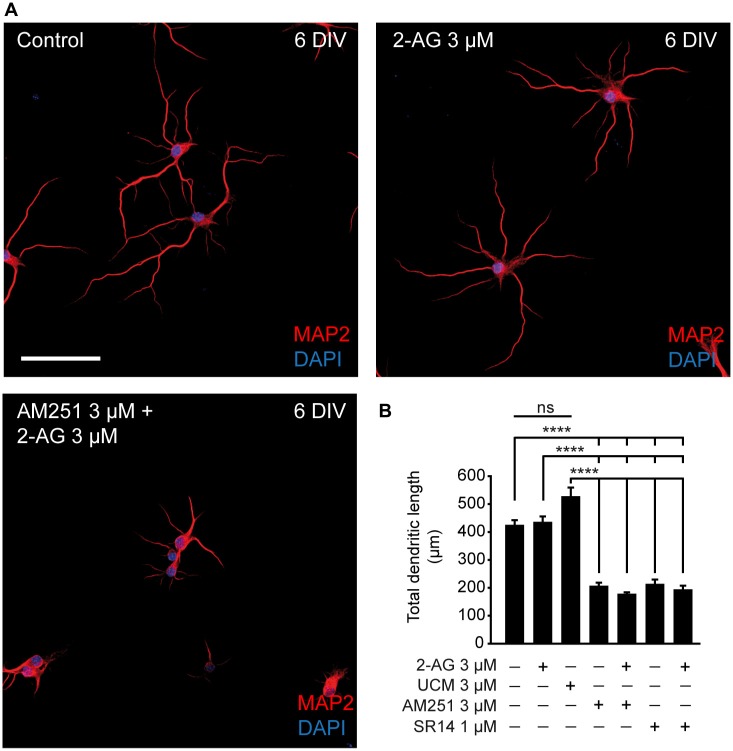
**CB1R activity is necessary for dendritic development. (A)** 6-DIV hippocampal neurons treated with CB1R antagonists, and/or agonist or treated with the MAGL inhibitor UCM03025. Drugs were first added after plating and treatment was repeated every other day. After 6 days in culture, neurons were stained with MAP2 antibody to detect the somatodendritic domain and DAPI for nuclei. Scale bar = 100 μm. **(B)** Bar plots representing the mean ± SEM of total dendritic length. Data were acquired from three independent experiments (30 neurons/experimental condition in each experiment). Kruskal-Wallis, Dunn’s multiple comparisons test. Adjusted *p* values: ****p < 0.0001; ns, non-significant.

Our results are in agreement with the increased density of axonal markers observed during the development of zebrafish treated with the CB1 agonist WIN55212-2 (Gilbert and Soderstrom, 2014). However, while these authors showed an increased density of dendritic proteins after CB1R activation during development (but not in adult), we did not detect CB1R expression in dendrites. In this regard, while somatodendritic CB1R visualization is difficult using immunocytochemical and/or immunohistochemical approaches, new evidence of functional CB1Rs in postsynaptic dendritic domains have been provided by electrophysiological (Bacci et al., 2004; Marinelli et al., 2009) and live imaging studies (Leterrier et al., 2006; Ladarre et al., 2015). To test whether CB1R had an effect on dendritic development, hippocampal neurons were cultured until 6 DIV and treated at 0, 2 and 4 DIV with 2-AG or CB1R antagonists alone or combined (Figure 2A). Unlike what was observed for axonal growth, neither 2-AG nor UCM-03025 treatments did significantly modify the total dendritic length (433.18 ± 22.75 μm for 2-AG and 524.8 ± 34.28 μm for UCM μm vs. 422.06 ± 20.25 μm in control neurons, Kruskal-Wallis, Dunn’s multiple comparisons test, Figure 2B). However, when neurons were treated with CB1R antagonists, total dendritic length was reduced to less than 50% of the control value (204.18 ± 14.06 μm for AM251 and 191.07 ± 16.56 μm for SR1141716A vs. 422.06 ± 20.25 μm in control neurons, Kruskal-Wallis, Dunn’s multiple comparisons test, *p* < 0.0001 Figure 2B). The same result was obtained when antagonists were applied in combination with 2-AG. Altogether, these data suggest a differential modulation of somatodendritic and axonal morphologies by CB1Rs: CB1R basal activity seems necessary for adequate dendrite development, while axon growth does not require CB1R activity, although application of exogenous 2-AG can potentiate axon growth.

### Cannabinoid Receptors and Axon Initial Segment Development

**Figure 3:**
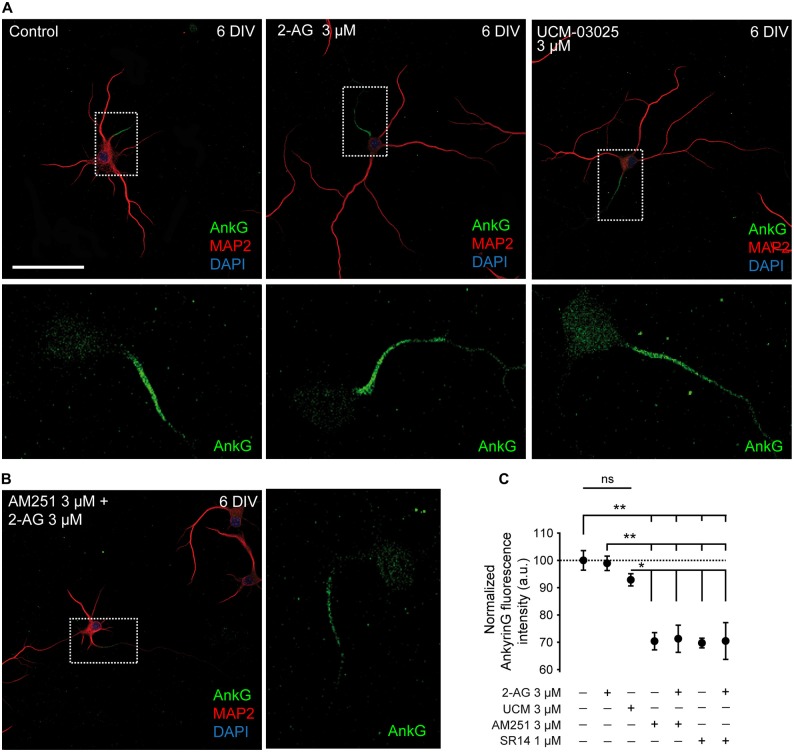
**CB1R inhibition decreases ankyrinG expression at the developing AIS. (A,B)** 6-DIV hippocampal neurons treated with 2-AG or UCM-03025 **(A)**, or 2-AG in combination with AM251 **(B)**. AIS domain stained for ankyrinG is included in a box and its magnification shown below **(A)** or in the right **(B)** for every image. Drugs were first added after cell plating and treatment was repeated every other day. After 6 days in culture, neurons were fixed and stained for MAP2 (red) to visualize somatodendritic compartment, and ankyrinG (AnkG, green) to visualize the AIS. All images were acquired using the same fluorescence parameters. Scale bar = 100 μm. **(C)** Bar plots representing the mean ± SEM of normalized AnkyrinG total fluorescence intensity along the AIS. Data were acquired from three independent experiments (30 neurons/experimental condition in each experiment). ANOVA, Bonferroni multiple comparisons test. Adjusted *p* values: **p < 0.01; *p < 0.05; ns, non-significant; a.u., arbitrary units.

Since theoretical mathematical models have shown that dendritic size and/or morphology influence AP onset and AIS plasticity (Eyal et al., 2014; Gulledge and Bravo, 2016), we hypothesized that the basal CB1R activity related to dendritic development described here could also influence AIS development. Thus, we investigated whether CB1R had an effect on AIS formation and maturation. For that purpose we analyzed the density of one of the main proteins of the AIS, ankyrinG, in different experimental conditions modulating both the activity and the expression of CB1Rs. First, we analyzed the possible role of CB1R on the formation and initial development of the AIS. We treated hippocampal neurons every other day (0, 2 and 4 DIV) until 6 DIV with 2-AG and CB1R antagonists alone or combined, as mentioned above for the analysis of dendrites (Figures 3A,B). As shown for dendritic development, 2-AG or UCM-03025 treatments did not modify the ankyrinG fluorescence signal compared to control neurons (100 ± 3.58% for control neurons vs. 98.93 ± 2.62% for 2-AG and 92.87 ± 2.24% for UCM, ANOVA, Bonferroni multiple comparisons test, Figure 3C). By contrast, CB1R inhibition with AM251 or SR141716A alone or in combination with 2-AG reduced the ankyrinG signal by ~30% (70.39 ± 3.16% for AM251, 69.73 ± 1.76% for SR141716A, 71.29 ± 4.97% for AM251 + 2-AG and 70.48 ± 6.73% for SR141716A + 2-AG vs. 100 ± 3.58% in control neurons, ANOVA, Bonferroni multiple comparisons test, *p* < 0.01 Figure 3C). These results clearly suggest that CB1R activity modulates AIS formation during early development *in vitro*, and that a basal CB1R activity is necessary for initial dendritic formation and AIS accumulation of ankyrinG.

**Figure 4:**
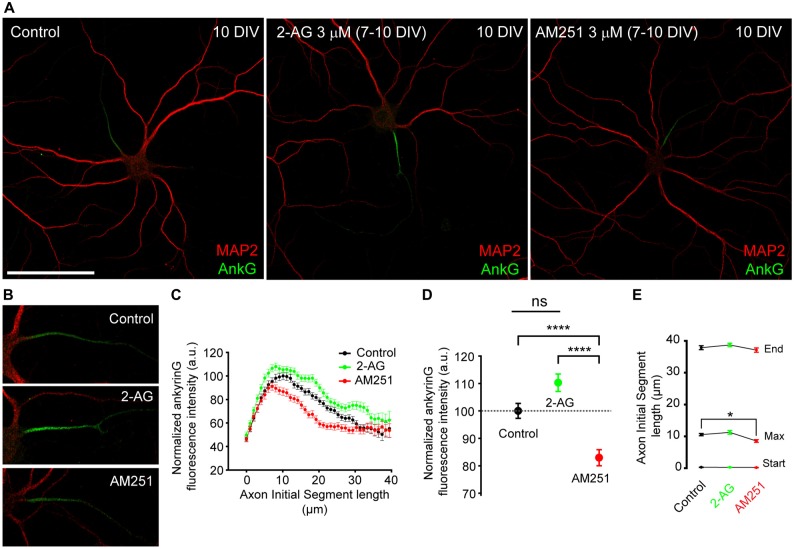
**CB1R inhibition, after dendrites formation, diminishes ankyrinG expression at the AIS. (A,B)** 10-DIV hippocampal neurons treated daily with cannabinoid receptor agonist 2-AG or CB1R antagonist AM251 from 7 to 10 days. High-magnification pictures of the AIS of each neuron are shown in **(B)**. Scale bar = 100 μm. **(C)** AnkyrinG fluorescence intensity profile along the AIS in control, 2-AG- or AM251-treated neurons. Data were acquired from three independent experiments (30 neurons/experimental condition in each experiment). **(D)** Normalized ankyrinG total fluorescence intensity at the AIS of neurons represented in **(C)**. **(E)** Graph representing the mean ± SEM of the start and end positions of the AIS, as well as, the position of the maximum fluorescence intensity at the AIS for each experimental condition. Kruskal-Wallis, Dunn’s multiple comparisons test. Adjusted *p* values: *p < 0.05; ****p < 0.0001, ns, non-significant; a.u., arbitrary units.

We then wondered whether CB1R activity could also participate in AIS maintenance and maturation at later stages of development after dendritic maturation has started. Thus we treated 7-DIV hippocampal neurons daily for 3 days, until 10 DIV, with 3 μM 2-AG or 3 μM AM251 (Figures 4A,B) and then we analyzed the expression of ankyrinG along the AIS (Figure 4C) and the total ankyrinG expression in the AIS (Figure 4D). As had been observed for dendrites, treatment with 2-AG did not significantly increase ankyrinG intensity in the AIS (110.28 ± 3.17% vs. 100 ± 2.72% in control neurons), whereas CB1 inhibition with AM251 decreased ankyrinG intensity by ~20% (82.94 ± 2.9%, Kruskal-Wallis, Dunn’s multiple comparisons test, *p* < 0.0001, Figure 4E). No change in AIS length or position was observed (Figure 4E). However, the maximum fluorescence intensity position shifted slightly and significantly by 2 μm towards the soma in AM251-treated neurons compared to control neurons (Kruskal-Wallis, Dunn’s multiple comparisons test, *p* < 0.05). In addition, no apparent changes in dendritic arbor were observed after 2-AG or AM251 treatments. Thus, while CB1R activity modulates the initial development of dendrites, at least *in vitro*, CB1R inhibition after 7 DIV does not affect the growth of dendrites, and is only necessary for AIS maturation.

**Figure 5:**
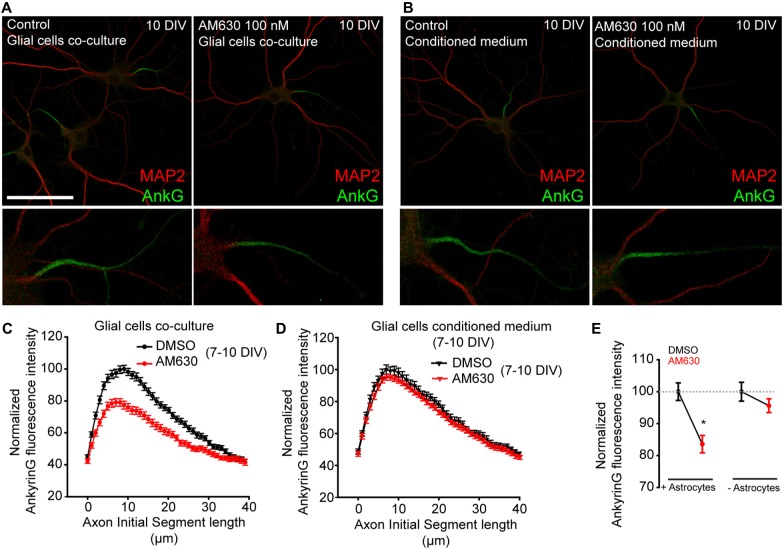
**Glial type-2 cannabinoid receptors (CB2Rs) modulation of AIS. (A,B)** 10-DIV hippocampal neurons treated daily with the CB2R antagonist AM630 from 7 to 10 days. Neurons were grown in the presence of a glial cell layer until 7 days (co-culture). Then, one group was treated in the presence of glial cells **(A)** and the second group was transferred to other plates containing only conditioned medium **(B)**. Neurons were stained with the somatodendritic marker MAP2 (red) and ankyrinG (green). Scale bar = 100 μm. **(C,D)** Graphs representing normalized ankyrinG fluorescence intensity profile along the AIS in control or AM630-treated neurons in the presence **(C)** or absence **(D)** of glial cells. **(E)** Graph representing the normalized total ankyrinG fluorescence intensity at the AIS of neurons represented in **(C,D)**. Data were acquired from three independent experiments (30 neurons/experimental condition in each experiment). Mann-Whitney Rank test. *p < 0.05.

One more point that needs to be considered is the fact that CB1R expression and activity in astrocytes (Navarrete and Araque, 2010) may be essential at these developmental stages for proper control of neuronal excitability. Indeed, CB1R activation in astrocytes can trigger the astrocytic release of neurotransmitters onto neurons (Gómez-Gonzalo et al., 2015). On the other hand, while CB2R expression in astrocytes and neurons in physiological conditions is controversial (Stella, 2010), this receptor has a role in embryonic and neural progenitors (Palazuelos et al., 2012). Thus, we tested whether CB2R activation by 2-AG or any possible non-CB1 effect of AM-251 on CB2R (Pertwee, 2005) could be responsible for AIS changes. First, we cultured 7-DIV hippocampal neurons for 3 days in the presence or absence of a layer of astroglial cells (Figures 5A,B). In the absence of glial cells, neurons were cultured in glial cell-conditioned medium. Neurons were treated daily for 3 days with the CB2R antagonist AM630 (100 nM) and then we analyzed ankyrinG intensity along the AIS (Figures 5C,D). Interestingly, the presence of a glial cell layer reduced ankyrinG maximal intensity by 20% when CB2 receptor was inhibited (83.55 ± 2.72% vs. 100 ± 2.75% in control neurons, Mann-Whitney Rank test, *p* < 0.05, Figure 5E). However, no change in ankyrinG intensity was detected in neurons treated in the absence of glial cells (95.61 ± 2.16% vs. 100 ± 2.95% in control neurons, Mann-Whitney Rank test, Figure 5E). Hence, while a neuron-specific CB2R effect on AIS maturation can be discarded, glial CB2Rs may also regulate the AIS.

**Figure 6:**
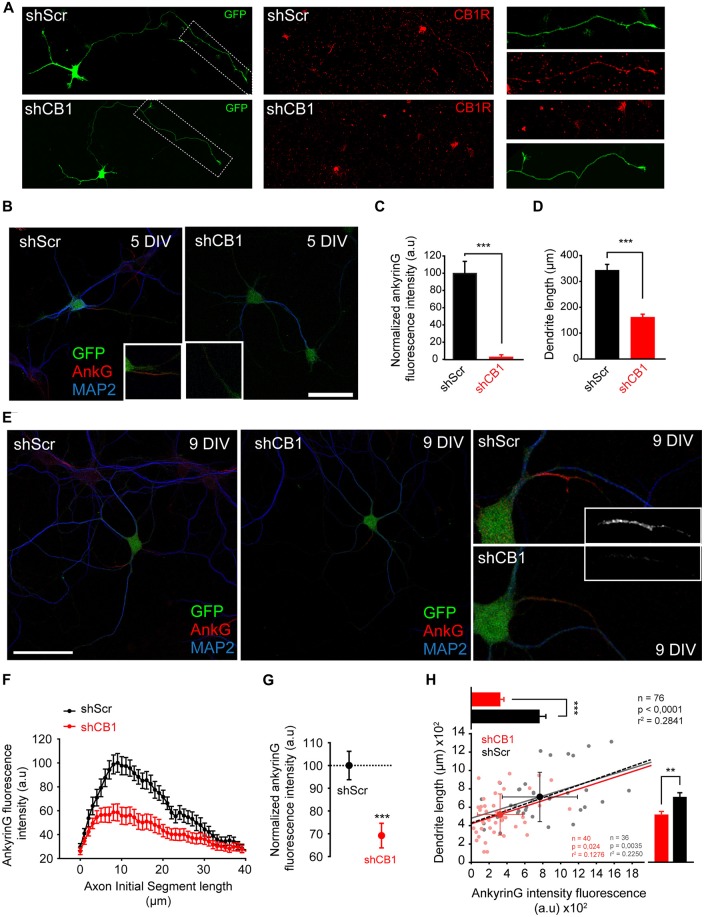
**Early CB1R suppression decreases ankyrinG concentration at the AIS and reduces dendritic length. (A)** 3-DIV hippocampal neurons nucleofected with scrambled interference RNA (shScr) or CB1R interference RNA (shCB1). Nucleofected neurons were identified based on GFP fluorescence. Surface CB1 receptor expression (red) was identified using a CB1R antibody that recognizes an extracellular epitope at the N-terminal domain. Note that CB1R staining was performed in living neurons at 37°C and neurons were rinsed quickly before fixation, generating the observed background. **(B)** Representative images of 5-DIV hippocampal neurons nucleofected with CB1R interference RNA (shCB1) or scrambled interference RNA (shScr). Neurons were stained with MAP2 antibody (blue) and ankyrinG antibody (red). Nucleofected neurons were identified based on GFP fluorescence. Insets show the AIS region. Note the strong reduction of ankyrinG staining and dendritic length in 5 DIV shCB1 neurons compared to shScr neurons. **(C,D)** Bar plots representing the mean ± SEM of normalized total AnkyrinG fluorescence intensity along the AIS **(C)** and total dendritic length of 5-DIV nucleofected neurons (**D**, *n* = 24, 13 shScr, 11 shCB1). **(E)** Representative images of 9-DIV hippocampal neurons nucleofected with CB1R interference RNA (shCB1) or scrambled interference RNA (shScr). Scale bar = 100 μm. The right panels show higher-magnification images of the AIS for the nucleofected neurons represented on the left panels. **(F)** AnkyrinG fluorescence intensity profile along the AIS of shScr- or shCB1-nucleofected neurons. Data were obtained from at least 40 neurons of each condition from three independent experiments. **(G)** Normalized ankyrinG total fluorescence intensity at the AIS for the neurons represented in **(B)**. Mann-Whitney Rank test *** *p* < 0.001. **(H)** Cell-to-cell analysis of the relationship between dendrite length and ankyrinG total fluorescence intensity at the AIS in 9-DIV nucleofected neurons. Scatter plot illustrating the significant correlation between ankyrinG intensity fluorescence (*x*-axis) and dendrite length (*y*-axis) of 76 nucleofected neurons (36 shScr, gray dots; 40 shCB1, light red dots). Regression lines and the *r*2 and *p* values for all neurons (black), for shScr neurons (gray) and shCB1 neurons (red) are indicated on the graph (Pearson product moment test). Bright colored dots represent the average (± SD) for the two groups. The bar plots shown on the right and top of the graph represent the mean (± SEM) for the cells plotted for each group, and significant statistical differences are represented. Mann-Whitney Rank test. ***p < 0.001, **p < 0.01. a.u., arbitrary units.

In order to rule out that ankyrinG reduction due to CB1R antagonist could be due to CB1Rs expressed by glial cells, we nucleofected hippocampal neurons with plasmids expressing the CB1R interference RNA (shCB1) or its corresponding scrambled RNA (shScr). We used previously validated shRNAs that were able to suppress ~50% of CB1R protein and mRNA expression in P19 cells (Diaz-Alonso et al., 2012). CB1R reduction in hippocampal neurons was confirmed by assessing CB1R expression in 3 DIV neurons nucleofected with shCB1 or shScr plasmids (Figure 6A) using a CB1R antibody that recognizes the extracellular N-terminal domain. CB1R signal was detected at the distal region of the axon only in shScr control neurons, while it was not detectable in shCB1 expressing neurons, confirming the validity of our plasmids. Then, we analyzed ankyrinG expression and dendrite length in nucleofected neurons kept for 5 DIV (Figure 6B). In 5-DIV shCB1 neurons, ankyrinG signal at the AIS was almost absent (2.46 ± 0.74% compared to 100 ± 13.73% shScr neurons, Mann-Whitney Rank test, *p* < 0.001, Figure 6C). Analysis of dendrite length in the same neurons showed a reduction in dendritic length of more than 50% in shCB1 neurons (159.88 ± 13.33 μm compared to 341.55 ± 24.35 μm in shScr neurons, Mann-Whitney Rank test, *p* < 0.001, Figure 6D). In order to confirm these results in neurons with a higher degree of dendritic development, we analyzed the data in 9-DIV nucleofected neurons (Figure 6E). shCB1 neurons showed a clear reduction of ankyrinG staining all along the AIS (Figure 6F), and a total ankyrinG fluorescence intensity 30% lower than in shScr control neurons (69.2 ± 5.4% vs. 100 ± 6.24% in shScr neurons, Mann-Whitney Rank test, *p* < 0.001, Figure 6G), in line with the results obtained previously with CB1R antagonists (Figure 3). This reduction was not associated to any change in position, length or maximum fluorescence position. Therefore, these experiments demonstrate that the effects of CB1R antagonists are due to neuronal CB1R inhibition. Next, we analyzed dendrite length in 76 of these neurons (36 shScr; 40 shCB1) and, as had been observed for 5-DIV nucleofected neurons (Figure 6D) and 6-DIV neurons treated with CB1R antagonists (Figure 2B), dendrite length was significantly reduced. While control shScr 9-DIV neurons had a mean total dendritic length of 710.31 ± 44.7 μm, mean dendritic length of shCB1 neurons was only 518.96 ± 35.38 μm (Figure 6H, bar plot on the right, Mann-Whitney Rank test, *p* < 0.01). To test a possible relationship between dendrites and AIS, we performed a cell-to-cell correlation of these two parameters in both conditions (Figure 6H). Dendritic length and AnkyrinG intensity were found to be positively correlated in the whole neuronal population (*p* < 0.001, *n* = 76), but also in neurons of each experimental condition taken separately (*p* < 0.005 for 36 shScr neurons and *p* < 0.01 for 40 shCb1 neurons). Moreover, a positive correlation was also obtained between ankyrinG fluorescence intensity and dendritic length in 5-DIV neurons (*p* < 0.0001, Pearson product moment test). Altogether these results confirm that functional neuronal CB1Rs contribute, likely through dendritic growth regulation, to ankyrinG accumulation at the AIS in hippocampal neurons at both early and late stages of development. Moreover, our results also show that CB1R-mediated changes in AIS composition and dendritic growth are correlated at initial dendritic development stages. Thus, a CB1 basal activity is necessary during very early neuronal development for AIS development and maturation. Further studies are necessary to understand which role pre- and post-synaptic CB1Rs may play on AIS maturation and plasticity within intact networks.

## Discussion

The ECS was initially characterized as a retrograde synaptic signaling system in the adult brain, where postsynaptically released eCBs bound to presynaptic CB1 receptors in GABAergic terminals (Katona et al., 1999; Ohno-Shosaku et al., 2001; Wilson and Nicoll, 2001). However, the classical view of the ECS in the CNS has dramatically changed over the last two decades, and in this new scenario the eCB system has been reported to regulate functions in many neuronal subtypes, in different subcellular locations, and to be associated with a plethora of down-stream modulators (Busquets Garcia et al., 2016; Lu and Mackie, 2016). Moreover, during CNS development the eCB system undergoes readjustments at multiple levels, showing the tremendous complexity of its effects on neuronal physiology (Katona and Freund, 2012).

**Figure 7:**
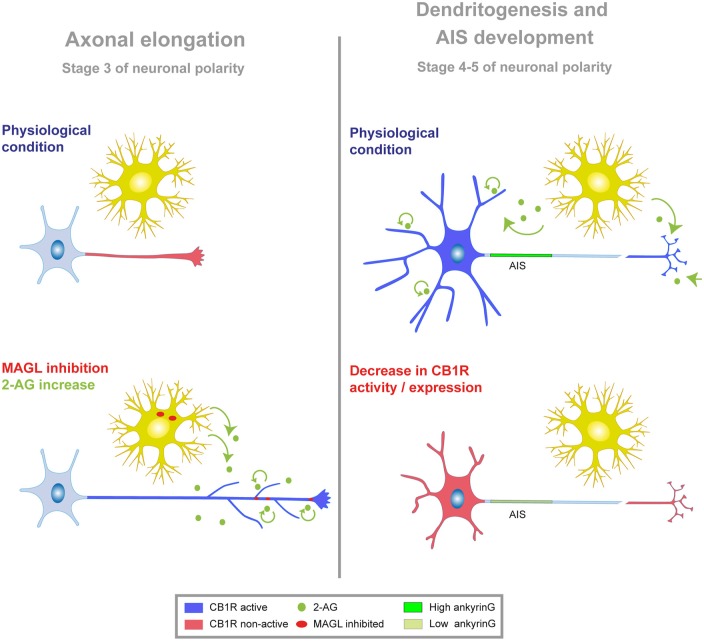
**Model of CB1R modulation of neuronal morphology and AIS.** Schematic showing the functional implication of CB1R activity at different stages of neuronal development *in vitro*. Neuronal developmental stages 1–5 have been defined previously by Dotti et al. (1988). Stage 3 is characterized by axonal polarization and elongation, while dendritogenesis and AIS development occurs from stages 4–5. In both cases, figures represent the effect of CB1R inhibition or suppression at stage 1, when neurons are not yet polarized. Left, during initial stages of neuronal polarization and axonal elongation, CB1Rs display low or negligible activity (red) in the axon. Exogenous or endogenous increase of 2-AG level (through MAGL inhibition, red dots; or exogenous application, green dots) promotes axonal elongation and branching through CB1R activation (blue). Evidence so far suggests that endogenous 2-AG may come from glial or neuronal cells. Right, at later stages of neuronal development (stages 4–5), CB1R activity (blue) in the dendrites and/or in the axons terminals and synapses contributes to dendritic and AIS development. Reducing CB1R activity or expression (red) impedes dendritic growth and reduces ankyrinG levels in the AIS.

In this study, we focused on the role of the eCB system during different stages of *in vitro* axonal, dendritic and AIS development. We show for the first time that neuronal CB1R basal activity indirectly contributes to AIS proteins accumulation during AIS initial development through the modulation of early developmental dendritic growth. Our data demonstrate that neuronal CB1Rs modulate axonal and dendritic morphology in very early stages of development. Moreover, our results demonstrate a correlated influence of these receptors on dendritic length and ankyrinG density at the AIS. In addition, we have identified a more modest role for glial CB2R in AIS modulation. These results suggest that a fine-tuned eCB signaling early in neuronal development acts as a modulator and coordinator of neuronal morphology and AIS composition (Figure 7).

### Modulation of Axonal and Dendritic Development by Cannabinoids

Our results show that CB1R is immunodetected in the distal part of the axon. However CB1R activity is not essential for axon establishment or the proper elongation of the axon at initial developmental stages, although its activation by 2-AG potentiates axonal growth and ramification in cultured hippocampal neurons. Several groups have suggested that CB1R antagonism/agonism can either promote or inhibit axon elongation and/or ramification depending on the agonist or antagonist used, the time of exposure, the age of culture and the type of neurons used (Gaffuri et al., 2012). Moreover, the strength of coupling of CB1Rs to downstream signaling pathways seems to also depend on the neuronal population considered: while CB1Rs are expressed at lower densities in glutamatergic neurons compared to GABAergic neurons, they are more strongly coupled to G-protein signaling in the first cell type (Steindel et al., 2013). These glutamatergic neurons represent 95% of the neurons in our primary cultures (Benson et al., 1994). Thus, differences between experimental models may partially explain the heterogeneity of data. In this study, we clearly demonstrate that an increase in 2-AG levels (exogenous or endogenous—induced by MAGL inhibition) potentiates axonal growth of hippocampal neurons *in vitro* in a CB1R-dependent manner. The question remains whether the effect of CB1R on axon elongation and ramification is direct or indirect. CB1R activation has been shown to induce the expression of trophic factors, such as BDNF, that promote axon growth and branching (Danzer et al., 2002; Derkinderen et al., 2003; Marsicano et al., 2003). Moreover, several studies showed a crosstalk between tyrosine kinase receptors and the eCB system in several neuronal subtypes: CB1R can form complexes and trans-activate TrkB receptors (Berghuis et al., 2005) or couple activated FGF receptors to an axonal growth response in cultured neurons (Williams et al., 2003). In addition, CB1R is also linked to other signaling pathways involved in neuronal differentiation and axonal growth (Keimpema et al., 2011; Galve-Roperh et al., 2013), such as the PI3K-Akt-GSK3 pathway (Ozaita et al., 2007) or JNK (Rueda et al., 2000).

Regarding dendritic development and unlike what happens during axonal development, triggering CB1R activation by 2-AG has no effect on dendritic elongation. However, our results point to a basal CB1R activity necessary for dendritic development, suggesting a CB1R differential regulation of axonal and dendritic development. Although our results show an axonal distribution of CB1R by immunofluorescence, CB1R may have a transient expression in dendrites after which somatodendritic endocytosis may contribute to target the receptor to the axon (Leterrier et al., 2006; McDonald et al., 2007). This axonal polarization process due to somatodendritic endocytosis has been described for other proteins in neurons (Garrido et al., 2001; Sampo et al., 2003). This differential regulation of axon and dendrites development by CB1R can be explained by differential basal activity of the receptor in each domain. CB1Rs are constitutively active in dendrites due to 2-AG local production, while axonal CB1Rs are not constitutively active (Ladarre et al., 2015). Local dendritic 2-AG production may explain why exogenous addition of 2-AG does not promote dendritic growth, while CB1R inhibition impairs dendritic elongation. On the other hand, the fact that axonal CB1Rs have no basal activity may explain why CB1R antagonists have no effect, and why only exogenous CB1R activation promotes axonal growth. This subcellular difference in CB1R activity may lead to differential inhibition of adenylyl cyclases and differences in cAMP and cGMP concentrations in both neuronal compartments. In fact, higher cAMP levels in axons are associated with axon formation and elongation, while dendritic development correlates with lower cAMP levels and higher cGMP levels (Shelly et al., 2010). Thus, the difference of CB1R activity and signaling between both compartments may explain the opposite results obtained with 2-AG and CB1R antagonists between axons and dendrites.

### Endocannabinoid System and Axon Initial Segment Formation and Maturation

Development of neuronal domains follow a well-defined time course in cultured hippocampal neurons (Dotti et al., 1988), where AIS initial formation and maturation take place after early axonal elongation and coincides with initial dendritic development. In this study, we demonstrate that functional neuronal CB1Rs participate in the regulation of AIS proteins composition, through the modulation of dendritic development. CB1R inhibition or knockdown reduced ankyrinG expression at the AIS, while CB1R activation had no significant effect on ankyrinG levels. Since CB1R location at the AIS has not been detected so far, we can hypothesize that axonal or dendritic CB1R activity is responsible for the changes in ankyrinG density we observed. In our low-density cultures and during early stages of development, neurons have not yet developed dendritic spines and neuronal connectivity is very low, making the hypothesis of a CB1R effect on dendrites the most plausible hypothesis to explain AIS changes during these stages. In fact, we demonstrate a significant positive correlation between dendritic length and ankyrinG levels in both control neurons expressing CB1R or neurons where CB1R expression was knocked down using RNA interference. In other words, our results show that a tonic CB1R activation would be critical for a proper initial dendrite growth, which modulate subsequent AIS development and maturation. This dendritic-AIS correlation only occurs when CB1R activity is impaired from very early stages of development. Nevertheless, CB1R inhibition at later stages of development (6 DIV, after dendrite maturation has started and neuronal connectivity increases) affects the AIS by reducing ankyrinG density. Thus, at later stages, we cannot rule out that reduced CB1R presynaptic activity may also participate in the modulation of AIS when dendritic development is not impaired by CB1R inhibition. Previous studies have shown a relationship between axonal and dendritic growth and ankyrinG density at the AIS. For example, impaired activity of the tubulin deacetylase HDAC6 reduces axonal and dendritic growth in early stages of development, and at the same time reduces AIS protein density (Kim et al., 2009; Tapia et al., 2010). Also, a recent study from the Südhof laboratory described a tight relationship between dendritic size, axonal growth and ankyrinG density at the AIS in neurons after L1CAM conditional deletion (Patzke et al., 2016). Moreover, a very recent study demonstrates that AIS distance to the soma in cortical layer 5 pyramidal adult neurons inversely correlates with dendritic complexity (Hamada et al., 2016), supporting the idea that dendritic morphology and AIS might be regulated in a coordinated manner in several neuronal types. Taking these studies and our results into account, change in dendritic morphology induced by CB1R lack of function is the more plausible hypothesis to explain AIS changes. However, we cannot rule out the existence of a direct effect on AIS due to unknown mechanisms.

These results raise the question of which mechanisms, and related molecules and receptors, are involved in the CB1R-mediated modulation of the AIS. CB1R signaling mechanisms are highly complex and diverse, and these receptors have many other effectors apart from their canonical targets (see “Introduction” Section). Somatodendritic CB1Rs play a key role in short-term regulation of intrinsic excitability through the activation of GIRK channels (Bacci et al., 2004; Marinelli et al., 2009) but also by regulating the baseline levels of the *I*_h_ current (hyperpolarization-activated cationic current) in pyramidal hippocampal neurons (Maroso et al., 2016). Moreover, presynaptic CB1Rs are widely involved in short and long-term plasticity events at inhibitory and excitatory synapses (Chevaleyre et al., 2006; Kano et al., 2009). Thus, secondary changes in AIS maybe attributed to CB1R mediated changes in intrinsic excitability and/or in synaptic strength. CB1R may exert these neuromodulatory effects cooperating with other receptors, such as glutamate receptors (Varma et al., 2001), or even modulating the release of neurotransmitters, such as glutamate (Gerdeman and Lovinger, 2001). For instance, a crosstalk between purinergic receptors and the ECS has been proposed. In fact, several groups have recently reported that the purinergic system may participate in cannabinoid-dependent synaptic modulation, thus demonstrating the existence of a crosstalk between these two families of receptors (Kovacs et al., 2011; Ievglevskyi et al., 2012). Similar to what has been observed for CB1Rs, purinergic receptors are absent from the AIS but are expressed in the somatodendritic domain and axon terminals (del Puerto et al., 2012; Shrivastava et al., 2013; Pougnet et al., 2014). Moreover, P2X7 receptors modulate the density of ankyrinG and sodium channels at the AIS (del Puerto et al., 2015). Whether the crosstalk between CB1R and P2X7 receptors is involved in AIS modulation remains elusive, however both are able to modulate G proteins (Howlett et al., 1998; del Puerto et al., 2012) and signaling molecules like GSK3 (Ozaita et al., 2007; del Puerto et al., 2012), which participates in AIS modulation (Tapia et al., 2013).

Regarding our results showing an astroglial CB2R-dependent modulation of the AIS, one study in a GFP-CB2R transgenic mouse has detected the expression of CB2R in microglia cells, but not in astrocytes or neurons (Schmöle et al., 2015). We assessed whether microglia cells were present in our glial cultures by detecting a specific microglial marker, Iba-1, and found only a few (or none) microglia cells that are unlikely to explain the observed effect. Nevertheless, it is still possible that cultured astrocytes express very low levels of CB2Rs, even though CB2Rs have only been detected in astrocytomas (Sánchez et al., 2001). Further studies and the development of tools for CB2R detection will be necessary to investigate the potential role of CB2R in the regulation of AIS and neuronal excitability in physiological and pathological conditions. In fact, CB2Rs have been detected in neurons and glial cells only in pathological conditions (Viscomi et al., 2009; Choi et al., 2013).

## Conclusion

Our results show that cannabinoid receptors play an important role in coordinating dendritic development and maturation of the AIS, which may influence future events during neuronal maturation. While CB1Rs have been thoroughly studied at presynaptic and postsynaptic sites, our study show that CB1R modulation of dendritic growth also modulates ankyrinG density, and therefore voltage-gated sodium channel density at the AIS. Further studies regarding cannabinoid receptors are necessary to understand the role the ECS may have in the regulation of AIS structure and plasticity, and its relation with the presynaptic and postsynaptic domains. Furthermore, understanding AIS modulation in response to brain disorders, diseases or injury also requires to consider changes in other neuronal compartments.

## Author Contributions

JJG and MT conceived and designed experiments. MT, JJG and AD performed experiments and data acquisition. MT, JJG and AD analyzed and interpreted data. MT and JJG wrote the manuscript. WZ, AP and MJB and MC contributed to acquiring and analyzing data. CG contributed to data comprehension. All authors read and approved the final manuscript.

## Conflict of Interest Statement

The authors declare that the research was conducted in the absence of any commercial or financial relationships that could be construed as a potential conflict of interest.
